# A machine learning approach for viral genome classification

**DOI:** 10.1186/s12859-017-1602-3

**Published:** 2017-04-11

**Authors:** Mohamed Amine Remita, Ahmed Halioui, Abou Abdallah Malick Diouara, Bruno Daigle, Golrokh Kiani, Abdoulaye Baniré Diallo

**Affiliations:** 1 Laboratoire de bioinformatique, département d’informatique, Université du Québec à Montréal, Montreal, P.O. Box 8888 Downtown Station, H3C 3P8 Qc Canada; 2 Pharmaqam Center, Université du Québec à Montréal (Québec), Montréal (Quebec), PO BOX 8888 Downtown Station, H3C 3P8 Canada

**Keywords:** Sequence classification, Prediction, Virus classification

## Abstract

**Background:**

Advances in cloning and sequencing technology are yielding a massive number of viral genomes. The classification and annotation of these genomes constitute important assets in the discovery of genomic variability, taxonomic characteristics and disease mechanisms. Existing classification methods are often designed for specific well-studied family of viruses. Thus, the viral comparative genomic studies could benefit from more generic, fast and accurate tools for classifying and typing newly sequenced strains of diverse virus families.

**Results:**

Here, we introduce a virus classification platform, CASTOR, based on machine learning methods. CASTOR is inspired by a well-known technique in molecular biology: restriction fragment length polymorphism (RFLP). It simulates, *in silico*, the restriction digestion of genomic material by different enzymes into fragments. It uses two metrics to construct feature vectors for machine learning algorithms in the classification step. We benchmark CASTOR for the classification of distinct datasets of human papillomaviruses (HPV), hepatitis B viruses (HBV) and human immunodeficiency viruses type 1 (HIV-1). Results reveal true positive rates of 99%, 99% and 98% for HPV Alpha species, HBV genotyping and HIV-1 M subtyping, respectively. Furthermore, CASTOR shows a competitive performance compared to well-known HIV-1 specific classifiers (REGA and COMET) on whole genomes and *pol* fragments.

**Conclusion:**

The performance of CASTOR, its genericity and robustness could permit to perform novel and accurate large scale virus studies. The CASTOR web platform provides an open access, collaborative and reproducible machine learning classifiers. CASTOR can be accessed at http://castor.bioinfo.uqam.ca.

**Electronic supplementary material:**

The online version of this article (doi:10.1186/s12859-017-1602-3) contains supplementary material, which is available to authorized users.

## Background

Genomic sequence classification assigns a given sequence into its related group of known sequences with similar properties, traits or characteristics. It is a fundamental practice in different research areas of microbiology yielding major challenges in comparative genomics. Accurate genomic sequence classification and typing could help to enhance the phylogenetics and functional studies of viruses [1]. They also help in determining pathogenicity, developing vaccines, studying epidemiology and drug resistance [1, 2]. Recent advances in DNA sequencing and molecular biology techniques provide an immense collection of genomic information. Such data volume raises challenges for genetic-based classification techniques. Three main approaches have been designed and implemented to classify different types of viruses based on their genomic sequence characteristics. The first is *sequence alignment-based* approach which is widely used, e.g. in similarity search methods (BLAST [3], USEARCH [4], etc.) and in pairwise distance based-methods (PASC [5], DEmARC [6], etc.). The second is *phylogenetic-based* approach. It is implemented in several tools, e.g. REGA [7, 8] and Pplacer [9]. The aim of these methods is to place an unknown sequence on an existing phylogenetic tree of a set of reference sequences. Each time a given sequence has to be classified, it is realigned with the set of reference sequences. Then, either a new phylogenetic tree is inferred or the given sequence is placed in the existing tree. The third is *alignment-free* approach including methods based on nucleotide correlations [10] and sequence composition [2, 11]. It transforms sequences or their relationships to feature vectors and then constructs a phylogeny, a statistical model or a machine learning model [12, 13]. These methods are reviewed in Vinga and Almeida [12], Mantaci et al. [14], Xing et al. [15] and Bonham-Carter et al. [13]. Restriction fragment length polymorphism (RFLP), a molecular biology technique [16], is used to type different virus strains [17–21]. Several algorithmic approaches have tackled theoretical and experimental problems related to the restriction enzyme data such as restriction mapping problem (see chap. 2 [22]), phylogeny estimation [23–25], SNP genotyping [26] and analysis of RFLP digitized gel images [27, 28]. However, large scale computational sequence classification based on the RFLP technique is not yet covered in literature. Due to the genetic polymorphism in DNA sequences, fragments resulting from enzyme digestions are different in terms of number and length between individuals or types. A set of restriction enzymes grounds a fragment pattern signature for each sequence. Therefore, similar sequences ought to have similar fragment patterns and thus similar restriction site distributions. This *a priori* knowledge could be used to build a machine learning model where sequences are represented by restriction site distributions as a feature vector and a class feature corresponding to a taxonomic level (genus, species, etc.). In this paper we introduce CASTOR, a machine learning web platform, to classify and type sequences. CASTOR integrates a new alignment-free method based on the RFLP principle. Our *in silico* method is independent of the sequence structure or function and is also not organism-specific. CASTOR is designed to facilitate the reuse, sharing and reproducibility of sequence classification experiments.

## Methods

### Overview of the approach

**Fig. 1:**
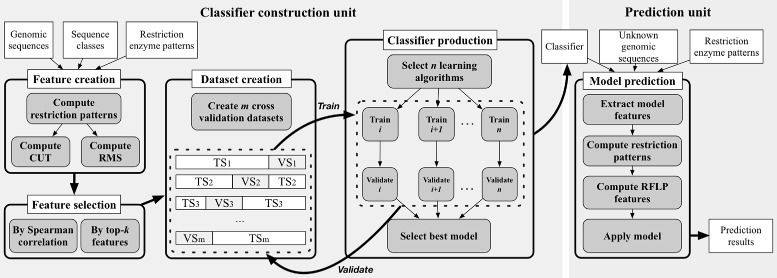
Overview of CASTOR kernel architecture. The kernel is composed of two main units (classifier construction and prediction). *White rectangles* represent input and output data; *grey and curved rectangles* represent processes. TS and VS are training set and validation set, respectively

In this paper, we propose an *in silico* approach to identify and classify viral DNA sequences based on their restriction enzyme sites using supervised machine learning techniques. Like other supervised learning approaches, the proposed one is divided into two main units (Fig. 1). The *classifier construction unit* builds and trains classification models (or classifiers). It requires a set of reference viral genomic sequences, their classes and a list of restriction enzyme patterns. It starts by creating a training set including a group of feature vectors. The latter is computed from the distribution of the restriction site patterns on the given DNA sequences and then refined by feature selection methods. A collection of learning classifiers are then trained and evaluated using 10-fold cross validation in order to choose the best classifier. The second unit (*prediction unit*) is intended to predict the classes or annotations of given viral sequences. The inputs of this unit are a classifier, a set of DNA sequences and the same list of restriction enzyme patterns used to train the classifier.

### Restriction fragment pattern-based features

Here, we propose a set of features simulating the outcome of the RFLP technique. From REBASE database [29], we extracted a list of 172 type II restriction enzymes and their recognition sites. Type II family cleaves (cuts) DNA sequences precisely on each occurrence of the recognition site. Then, the restriction digestion of DNA sequences is computationally simulated. In order to build a training set, for a sequence *s* and enzyme *z* we compute two metrics representing the distribution of the digested fragments: the number of cuts of the enzyme (*C* *U* *T*(*s*,*z*)) and the root mean square of digested fragment lengths (*R* *M* *S*(*s*,*z*)) calculated as

1 $$ RMS(s,z) =\sqrt{\frac{1}{n}\sum_{i=1}^{n}l_{i}^{2}}  $$

where *n* is the number of fragments (*C* *U* *T*(*s*,*z*)+1) and *l*_*i*_ is the length of the *i* ^*th*^ fragment in linear genomes. For circular genomes *n*=*C* *U* *T*(*s*,*z*). Other metrics could be easily computed from the fragment digestion to construct the feature vectors.

### Feature selection methods

The selection of an optimal subset of features improves the learning efficiency and increases the predictive performance. Feature selection techniques reduce the learning set dimension by pruning irrelevant and redundant features. Two relevant methods of feature reduction are provided. The first method (*topAttributes*) ranks the features according to their information gain [30] and selects a subset of top-*k* features. Information gain estimates the mutual information between a feature and the target class. The second method (*correlation*) uses the Spearman’s rank correlation coefficient to construct a set of uncorrelated features. The correlation coefficient between two feature ranking vectors *u* and *v* of size *n* is computed as follows:

2 $$ \rho=1-{\frac{6\sum_{i=1}^{n}(u_{i} - v_{i})^{2}}{n(n^{2} - 1)}}.  $$

A two-tailed *p-value* is computed to test the null hypothesis which states that two feature vectors are uncorrelated. In order to remove one of the two correlated features, two strategies could be used: discarding the feature with the largest sum of absolute correlation coefficients or the one with the smallest information gain score.

### Learning and evaluation

We explored three types of classifiers: (1) symbolic methods (C4.5 decision tree (J48) [31] and random forests (RFT) [32]), (2) statistical methods (naive Bayes classifier (NBA) [33, 34], support vector machine (SVM) [35] and K-nearest neighbors (IBK) [36, 37]) and (3) ensemble methods (Adaboost (ADA) [38] and Bagging (BAG) [39] both combined with J48); see Additional file 1: Table S1 for more details. A 10-fold cross-validation strategy is used to assess the performance of the trained classifiers. Performance measures are weighted according to the number of instances and computed for the overall classification. The performance measures are:

3) (4) (5) (6 $$\begin{array}{*{20}l} TPR &= TP/(TP + FN),\\ FPR &= FP/(FP + TN),\\ Precision &= TP/(TP + FP),\\ F-measure &= \frac{2 \times TPR \times Precision}{TPR + Precision}. \end{array} $$

where *TP*, *TN*, *FP*, and *FN* are the number of true positive, true negative, false positive and false negative predictions, respectively. *TPR* and *FPR* are the true positive rate and the false positive rate, respectively. We used Weka data mining program to perform the training and the evaluation [40].

To include a negative class in the training sets, two approaches could be used. First, provide manually constructed negative class from collected relevant data. Second, build it with the provided negative class generator. This generator constructs altered sequences data from a sampling with replacement of the positive set sequences. To alter the sampled sequences, we reshape the RFLP length distribution of the training set by randomly shrinking, expanding or keeping unchanged the length of the sampled sequences. Then, each sequence is randomly shuffled while preserving k-mer counts.

### Datasets

In this study, we applied our approach to a wide range of viruses. We selected one dsDNA virus (human papillomavirus (HPV)), one dsDNA-RT virus (hepatitis B virus (HBV)) and one ssRNA-RT virus (human immunodeficiency virus type 1 (HIV-1)). (1) HPVs have a circular double stranded DNA genome of ∼8000 bp and belong to five genera (Alpha, Beta, Gamma, Mu and Nu). HPVs belonging to a genus share over 53% identity of their complete genomes and ones in the same species level share over 62% of identity [41, 42]. We assessed the performance of HPV classification in the genus and species taxonomic levels. At the species level, we selected only the Alpha HPV genus representing the most abundant and diverse genomes in databases. It is divided into thirteen species (Alpha 1–11, Alpha 13–14). Unfortunately, some HPV genera (Mu and Nu) and Alpha HPV species (1, 5, 8, 11 and 13) were underrepresented and were therefore discarded. (2) HBV genomes are smaller (3200 bp) and are circular partly double stranded DNA. HBVs are classified into eight genotypes (A–H) with at least 8% divergence among their genomic sequences [43]. We evaluated the performances of our method for the genotyping of HBV strains. HPV and HBV complete genome sequences were downloaded from the NCBI RefSeq database [44]. The taxonomic annotations were extracted from the NCBI Taxonomy database [44]. (3) HIV-1 genomes have two copies of positive-sense single-stranded RNA with ∼9700 bp. Phylogenetically, HIV-1 strains are divided into four groups: M, N, O and P [45, 46]. M group strains are worldwide prevalent. They are categorized into pure subtypes (A–D, F–H, J and K) and recombinant forms (up to 70 CRFs and URFs). Genetic variations among subtypes are about 20–30% for *env* gene, 7–20% for *gag* gene and 10% for *pol* gene [47]. For HIV-1 classification, we studied complete genomes (CGs) and fragments covering *pol* gene from the position 2253 to 3554 with respect to HXB2 reference sequence and having a minimum size of 1 Kbp (*pol* fragments). HIV-1 sequences were extracted from the Los Alamos HIV sequence database (http://www.hiv.lanl.gov/). For all the datasets, only complete, curated and well-annotated sequences were selected. Moreover, each class ought to have an adequate number of genomic sequences in order to have a representative genetic diversity.

### Simulation studies

Raw viral sequence datasets, described above, were class-size imbalanced, i.e., the difference in the number of genome sequences belonging to each class was relatively large. Generally, epidemiological studies are conducted on host-specific viruses (human, cattle, etc.) with the highest prevalence and pathogenicity [48, 49]. This leads to more data for some groups of viruses over others. Usually, training standard classifiers on imbalanced datasets affects their performance (mainly sensitivity and specificity) and misleads the interpretation of their accuracy [50, 51]. Under-sampling majority class approach has been shown to perform well [52] and could be used with standard algorithms. Hence, from each previous dataset, we randomly performed under-sampling, without replacement, of the larger classes to have relatively the same sizes as the other classes. In order to identify the best parameters of the classifiers, we randomly sampled 10 datasets for each of the HPV genera, HPV Alpha species, HBV genotypes, HIV-1 M subtypes CGs and HIV-1 M subtypes *pol* fragments data. For each obtained sample, we performed a 10-fold cross-validation study with different classifiers built as follows. We constructed all the combinations of the two metrics (*CUT* and *RMS*), the two feature selection methods (*topAttributes* and *correlation*) and the seven learning algorithms. This construction yielded 28 *c* *o* *m* *b* *i* *n* *a* *t* *i* *o* *n* *s*∗10 *d* *a* *t* *a* *s* *e* *t* *s*=280 *e* *x* *p* *e* *r* *i* *m* *e* *n* *t* *s* for each virus classification.

## Results and discussion

The Results section is divided into four parts: first, we show how the RFLP signatures are suitable for viral classification; second, we assess the performance of several competing classification algorithms on different virus datasets; third, we compare the prediction made by CASTOR against widely used methods for HIV-1 datasets, one of the most difficult to classify and fourth, we present the CASTOR web platform.

### Classification with RFLP signatures in virus families

**Fig. 2:**
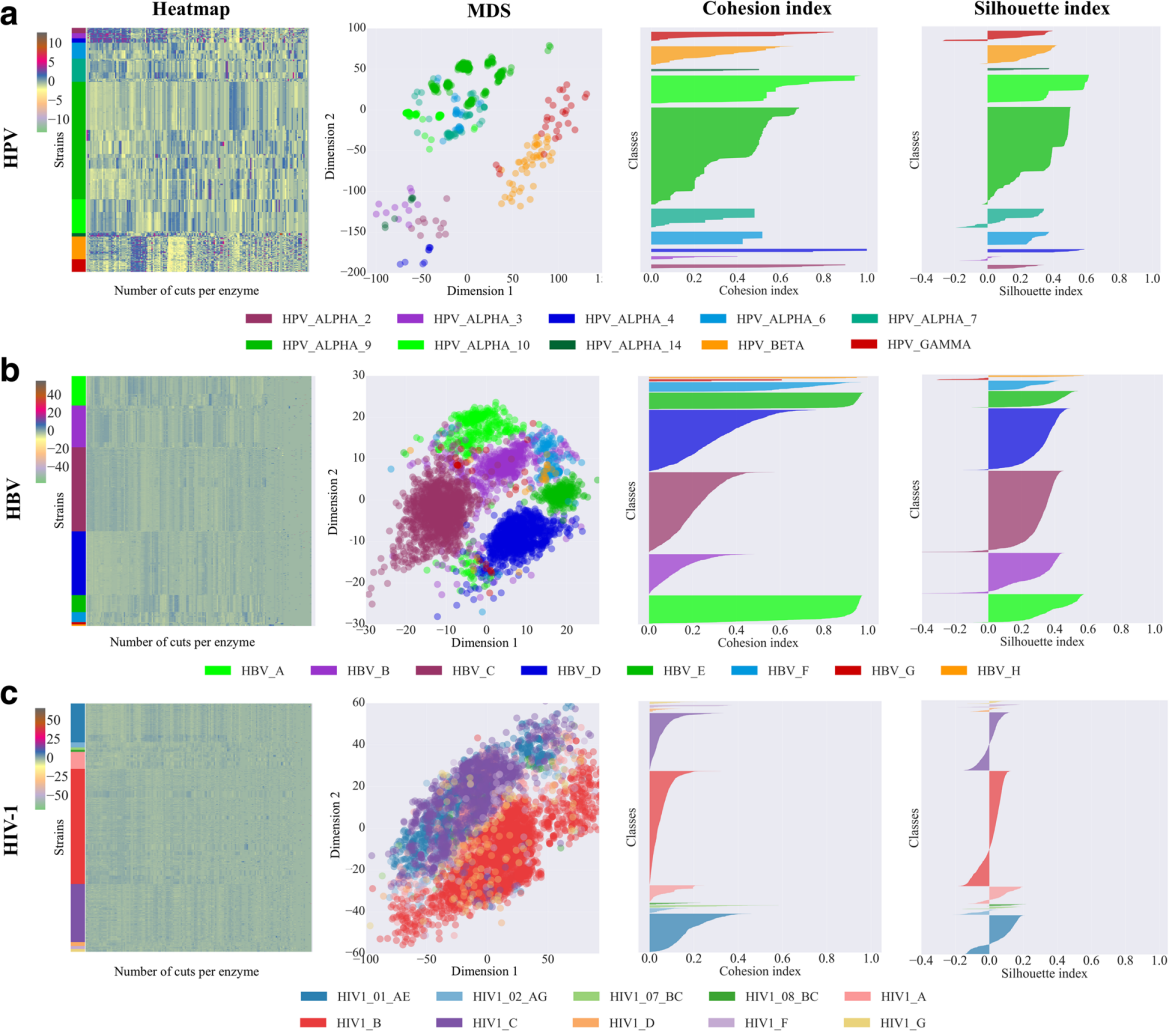
Class cohesion of three virus datasets. The *four columns* illustrate the separability and compactness of three virus complete genomes datasets based on 172 restriction enzyme cuts. The *first column* shows heatmaps of *CUT* clustered by x-axis. The samples in the y-axis are grouped by studied classes followed by intra-class clusterings. The *second column* shows MDS of the *CUT* distances between samples. The *third and fourth column* represent, respectively, the *Cohesion* and *Silhouette* indexes of the classes. **a** Classes in HPV are Alpha species, Beta and Gamma genera. **b** Classes in HBV are A-H genotypes **c** Classes in HIV-1 are M pure subtypes and CRFs

Figure 2 highlights the natural RFLP cuts in the collected HPV, HBV and HIV-1 datasets. The second column of the figure shows the multidimensional scaling (MDS) plot of the first two dimensions of distances between the feature vectors of the genomes. The separation between the different HPV genera (Fig. 2 a) could approximatively be drawn, which is partly the case for the HPV species. The *Cohesion* [41] and *Silhouette* [53] indexes allow to measure the compactness and separability of classes. Here, both indexes show moderate values (between 0.2 and 0.8 for *C* *o* *h* *e* *s* *i* *o* *n* *i* *n* *d* *e* *x* and –0.2 to 0.7 for *S* *i* *l* *h* *o* *u* *e* *t* *t* *e* *i* *n* *d* *e* *x*) indicating that the classes are not well distinct. Several instances could be mislabeled or share the same RFLP cut patterns with other classes. This results in low or negative values of *S* *i* *l* *h* *o* *u* *e* *t* *t* *e* a in HPV Alpha 3, 7 and HPV Gamma (Fig. 2 a). With CASTOR, the best HPV Alpha Species classification obtains a *TPR* of 0.992 and *FPR* of 0.002 in 10-fold cross-validation analyses of 118 instances (see Table 1). The power of RFLP cuts in classification of viruses could be observed in HBV genotypes heatmap (see Fig. 2 b). HBV highlights three genotypes (A, E and F) with *C* *o* *h* *e* *s* *i* *o* *n* *i* *n* *d* *e* *x* *e* *s* for most instances above 0.7 indicating very coherent classes. But B and C genotypes have values between 0.1 and 0.6. The *S* *i* *l* *h* *o* *u* *e* *t* *t* *e* *i* *n* *d* *e* *x* plots show several instances of B, C, E and G genotypes that have an striking disagreement with their assigned classes (*S* *i* *l* *h* *o* *u* *e* *t* *t* *e* *i* *n* *d* *e* *x*<−0.1). Even with these constraints, CASTOR achieves the genotyping of 230 HBV instances with *TPR* of 0.996 and *FPR* of 0.001 according to a 10-fold cross-validation study (see Table 1). The HIV-1 cut site patterns have more variability among pure subtypes and CRFs (Fig. 2 c). Likely, the MDS plot shows a moderate subtype clustering for the main HIV-1 subtypes. But this clustering is not well separated compared to HPV and HBV. This variability among classes is reflected in low values of the *C* *o* *h* *e* *s* *i* *o* *n* *i* *n* *d* *e* *x* (≤0.4). All, suggesting either variability, noise or mislabelling. For instance, >30*%* of HIV-1 B and HIV-1 C instances tend to have RFLP cut patterns of other subtypes (negative *S* *i* *l* *h* *o* *u* *e* *t* *t* *e* *i* *n* *d* *e* *x* *e* *s*). With CASTOR, the subtyping of HIV-1 group M within 18 main subtypes was assessed for 597 instances with a *TPR* of 0.983 and *FPR* of 0.001.

**Table 1:** CASTOR best accuracies on the classification of five datasets

Group of virus	Organism	Classification	# of classes	# of instances	*TPR*	*FPR*	*F-measure*	Classifier ID
I (dsDNA)	HPV	Genera	3	125	0.992	0.005	0.992	PMSHPV01
		Alpha species	8	118	0.992	0.002	0.992	PMSHPV02
VII (dsDNA-RT)	HBV	Genotypes	8	230	0.996	0.001	0.996	PMSHBV01
VI (ssRNA-RT)	HIV-1	Groups	4	76	1.000	0.000	1.000	PMSHIV01
		M Subtypes	18	597	0.983	0.001	0.983	PMSHIV02

This table contains the best results of the experimental study performed on the different datasets. The evaluation measures are obtained with 10-fold cross-validation analysis. The column Classifier ID contains the corresponding models available in CASTOR platform

Previously, it has been clearly shown that RFLP has a power for classification in several viruses such as HPV [17, 18], HBV [20] and HIV [19]. But these studies are mostly limited to two to five classes. To the best of our knowledge, our study constitutes the first large scale and multi-class analyses of RFLP cut for classification. It provides the basis to explore large and various types of classifications, in particular those based on machine learning methods.

### Machine learning classifier tuning and performance

The CASTOR platform relies on machine learning methods for the classification of viruses based on RFLP signatures of nucleotide sequences. The platform is detailed in the CASTOR web platform section. Three important parameters constitute the kernel of each CASTOR classifier: a metric, a feature selection method and a learning algorithm. To assess the different combination of the models, we performed a 10-fold cross-validation of the 280 experiments associated to each of the five datasets (HPV genera, HPV Alpha species, HBV genotypes, HIV-1 M subtypes CGs and HIV-1 M subtypes *pol* fragments). From the overall results of the five virus classifications, it is not obvious to distinguish the best candidate between *CUT* and *RMS* metrics. In the genotyping of HBV, *CUT* performs better than *RMS* (*p-value* = 0.0012, Wilcoxon/Kruskal-Wallis test) while in the HPV genera and species classifications *RMS* performs better than *CUT* (*p-values* 5.00E-03 and 0.0293, respectively; Wilcoxon/Kruskal-Wallis test) (Additional file 1: Figure S1). However the mean of weighted *F-measures* for both methods is in all cases ≥0.906 (with a minimum of 0.793 and a maximum of 0.996). The same analyses were performed on HIV-1 CGs and *pol* fragments. *CUT* performs slightly better than *RMS* in both datasets when comparing the mean of weighted *F-measures* (*p-values* 0.0213 and 0.0237 for CGs and *pol* fragments, respectively; Wilcoxon/Kruskal-Wallis test). Due to the variability of HIV-1, the mean of weighted *F-measures* falls to 0.857 in CGs and 0.793 in *pol* fragments (Additional file 1: Figure S1). Hence for the remaining of our study, we will fix the RFLP metric according to its performance on the corresponding datasets.

**Fig. 3:**
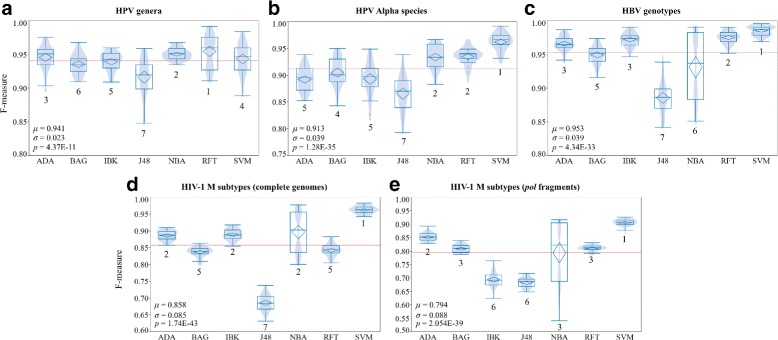
Learning algorithm evaluation on five datasets. This figure illustrates the *F-measure* distribution (boxplot) of seven learning algorithms on the prediction of **a** HPV genera, **b** HPV Alpha species, **c** HBV genotypes, **d** HIV-1 M subtypes with complete genomes **e** HIV-1 M subtypes with *pol* fragments. HPV and HBV datasets are complete genomes. The number below each boxplot corresponds to the statistically discriminative rank of the algorithms. The ranking is performed with paired Student’s t test. *μ*, *σ* are the mean and the standard deviation of the overall *F-measures*, respectively. *p* is the *p-value* of the statistically significance of the weighted *F-measure* mean differences among the algorithms computed with the Wilcoxon/Kruskal-Wallis test

Additional file 1: Figure S2 presents the comparative analyses of the two feature selection methods (*correlation* and *topAttribute*) in the 280 experiments for each dataset. The mean of weighted *F-measures* of the two feature selection methods are not statistically different in all datasets (based on the Wilcoxon/Kruskal-Wallis test). In fact, the results of the two methods are correlated for the three viruses with the Spearman’s rank correlation coefficient ranging between 0.772 and 0.968 (see Additional file 1: Figure S4). In these simulations, the seven learning algorithms have various performances according to the different datasets. The algorithm J48 has the worst weighted *F-measure* values (see Fig. 3). However, its performance improves when combined with RFT or BAG algorithms. In general, SVM performs better in four of five datasets with mean of weighted *F-measures* > 0.906 and ranks number one in HPV Alpha species, HBV genotypes and HIV-1 subtypes classifications and four in HPV genera classification. It is followed by RFT, NBA and IBK. However, RFT and NBA are affected by a large variance (Fig. 3). These rankings are clearly observable on Additional file 1: Figure S3 and Figure S4 presenting respectively the correlations *CUT*/*RMS* and *topAttribute*/*correlation* grouped by algorithms. While most algorithms have similar performance with *CUT* or *RMS*, Naive Bayes surprisingly performs better with *CUT*.

### Assessing the performance CASTOR on HIV-1 data

#### CASTOR exhibits high accuracy for different HIV-1 classification

Table 2 highlights CASTOR prediction accuracies on five CG and seven *pol* fragment HIV-1 classifications. For each dataset, the best performing models (classifiers) have been identified according to a 10-fold cross-validation analysis. The *F-measure* of the best classifier for the HIV-1 groups M, N, O and P indicates that all the sequences are correctly classified (for CGs and *pol* fragments). For the prediction of the main HIV-1 pure subtypes as well as CRFs, *F-measures* are above 0.971 (with *F* *P* *R*≤0.003) for both CGs and *pol* fragments when the pure subtypes and CRFs are separate models. When combining pure subtypes and CRFs, the *F-measure* still remains above 0.971 for CGs but it drops to 0.919 when the classes are balanced to 30 instances per class or 0.962 for 200 instances per class. It appears that the CASTOR models are underperforming when we try to predict between pure subtypes and CRFs (*F-measures* of 0.795 and 0.885 for CGs and *pol* fragments, respectively).

**Table 2:** Evaluation of HIV-1 classification with CASTOR

	Classification	# of classes	# of instances	[min - max] instances/class	*TPR*	*FPR*	*F-measure*	Classifier ID
Complete genomes	Groups (M, N, O and P)	4	76	[4 – 32]	1.000	0.000	1.000	PMVHIVGC01
	Pure subtypes	6	189	[30 – 36]	0.995	0.001	0.995	PMVHIVGC02
	CRFs	12	234	[10 – 30]	1.000	0.000	1.000	PMVHIVGC03
	Pure subtypes and CRFs	18	423	[10 – 36]	0.981	0.001	0.981	PMVHIVGC04
	Pure subtypes vs CRFs	2	200	[100 – 100]	0.795	0.205	0.795	PMVHIVGC05
*pol* fragments	Groups (M, N, O and P)	4	94	[4 – 45]	1.000	0.000	1.000	PMVHIVPL01
	Pure subtypes	6	1800	[300 – 300]	0.983	0.003	0.983	PMVHIVPL02
	CRFs	16	480	[30 – 30]	0.971	0.002	0.971	PMVHIVPL03
	CRFs	6	1200	[200 – 200]	0.993	0.001	0.993	PMVHIVPL04
	Pure subtypes and CRFs	23	690	[30 – 30]	0.920	0.004	0.919	PMVHIVPL05
	Pure subtypes and CRFs	12	2400	[200 – 200]	0.962	0.003	0.962	PMVHIVPL06
	Pure subtypes vs CRFs	2	200	[100 – 100]	0.885	0.115	0.885	PMVHIVPL07

This table contains the TPR, FPR and *F-measure* of 12 HIV-1 classifications obtained with 10-fold cross-validation analysis. For each classification, the number of corresponding classes and instances are given. The range [min-max] indicates the interval of instance frequencies per class used during the training of each model. The column Classifier ID contains the corresponding models available in CASTOR platform

#### Comparing COMET, REGA and CASTOR

**Fig. 4:**
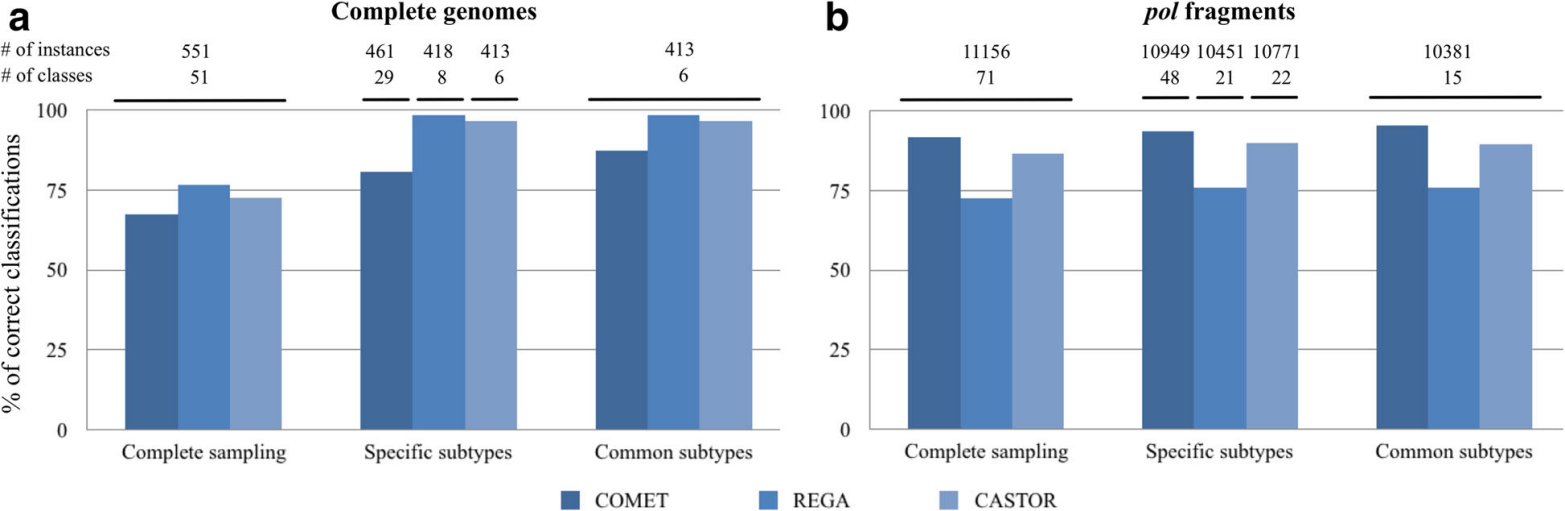
Performance of CASTOR with COMET and REGA predictors on HIV-1 datasets. The panels **a** and **b** show the percentage of correct classifications for HIV-1 complete genomes and HIV-1 *pol* fragments, respectively. The number of instances and the associated classes for each sampling is presented above the panels. Complete sampling corresponds to 10% of Los Alamos HIV data selected randomly. In specific subtypes sampling, the predictors are assessed against their trained classes. In common subtypes sampling, the predictors are assessed against the intersection of the classes of the three trained predictors

Next, we compared the performance of CASTOR against the most powerful and widely used HIV-1 specific predictors namely COMET [2] and REGA version 2.0 [7, 8] (Fig. 4). These comparisons are based on CG as well as *pol* fragment data. It is important to notice that these programs are fixed and do not allow neither any change on the trained classes nor new training samples. Here the actual training of COMET and REGA includes respectively 55 and 22 classes for either CG or *pol* fragments. To avoid under-represented classes, CASTOR was trained on 18 classes for CGs and 28 classes for *pol* fragments (models are available under the classifier IDs PMSHIV02 and PMSHIV03, respectively). We performed three comparisons (see Fig. 4). The first, named *complete sampling*, assesses the performance of each method on 10 percent of randomly sampled Los Alamos HIV data. This sampling permits to assess the performance of the predictors to fit realistic data with unknown classes. The second, named *specific subtypes*, focuses, for each method, only on the corresponding trained subtypes. The third, named *common subtypes*, compares the performance of the methods on the intersection of the 3 trained subtypes. This strategy is used due to the fact that the training of COMET and REGA cannot be changed. Thus, it is difficult to adapt or perform other classification studies or larger benchmark analyses. Figure 4 shows that for CGs, REGA performs the best followed by CASTOR and for *pol* fragments COMET outperforms, followed again by CASTOR. In the two types of data, when not performing the best, REGA or COMET performance drops drastically by more than 10% and ranks at the third position (Fig. 4). Meanwhile CASTOR ranks second in both two types of data. With CGs, CASTOR obtains a correct classification of 72.41% against the sampling of Los Alamos HIV data when REGA obtains 76.77%. But when testing predictors on their trained classes, the percentage of correct classification drastically increases to 98.33 and 96.61% respectively for REGA and CASTOR. This result remains almost the same when comparing only the common trained classes among the three predictors (Fig. 4). These common classes cover 75 and 93% of the overall instances of the sampling of CGs and *pol* fragments, respectively. The mean *TPR* of CASTOR is higher than 0.950 in the case of either pure subtypes or CRFs. The *TPR* of REGA drops to 0.835 when assessing CRFs and remains almost perfect for pure subtypes (Table 3). In *pol* fragments, COMET outperforms CASTOR and REGA in all comparisons. Applying the three methods, COMET, REGA and CASTOR, on 10% random sampling of Los Alamos HIV data, the percentages of correct classification were 91.74, 72.48 and 86.64%, respectively. This result is confirmed when comparing only the common trained classes where COMET reaches 95.57% and CASTOR 89.51%. Note that REGA could not perform higher than 76% and has a mean *TPR* of 0.953 for pure subtypes competing with COMET. In CRF instances, COMET and CASTOR obtain almost an equal mean of *TPR* around 0.930 (Table 4). REGA cannot perform well in CRF classification and has a mean of *TPR* equal to 0.570. CASTOR has higher *FPR* values compared to the two other programs in overall classifications. This fact is not surprising since REGA and COMET are specifically tuned to predict HIV data. Their predictions with lower scores tend to be discarded or ambiguous. For instance, COMET has 32% of its CG predictions that are unassigned as well as 5% of its *pol* fragment predictions. Hence, these numbers are higher than the false positive values of CASTOR, but they are not included in the *FPR* computation. However, it will be interesting to include in CASTOR a threshold of inclusion of a given sequence into a class. This could help reducing the *FPR* but it would require deeper analyses. It also should be associated to the *open-set* classification problem that is beyond the scope of this paper.

**Table 3:** Performances of HIV-1 predictors on complete genome classification

			COMET			REGA			CASTOR		
		# of instances	*TPR*	*FPR*	*F-measure*	*TPR*	*FPR*	*F-measure*	*TPR*	*FPR*	*F-measure*
CRFs	HIV1_01_AE	100	0.960	0.000	0.980	0.970	0.000	0.985	1.000	0.000	1.000
	HIV1_02_AG	10	0.900	0.000	0.947	0.700	0.000	0.824	0.900	0.007	0.818
	Mean		0.930	0.000	0.964	0.835	0.000	0.905	0.950	0.004	0.909
Pure subtypes	HIV1_A	100	0.660	0.000	0.795	0.990	0.000	0.995	0.940	0.000	0.969
	HIV1_B	100	0.910	0.000	0.953	1.000	0.000	1.000	0.960	0.003	0.975
	HIV1_C	100	0.970	0.000	0.985	1.000	0.000	1.000	0.970	0.003	0.980
	Mean		0.847	0.000	0.911	0.997	0.000	0.998	0.957	0.002	0.975

This table contains TPR, FPR and *F-measure* of COMET, REGA and CASTOR on the prediction of HIV-1 M pure subtypes and CFRs complete genomes. The shown classes belong to the common subtypes sampling. The CASTOR model used in this evaluation is PMSHIV02

**Table 4:** HIV-1 predictor performances on *pol* fragment classification

			COMET			REGA			CASTOR		
		# of instances	*TPR*	*FPR*	*F-measure*	*TPR*	*FPR*	*F-measure*	*TPR*	*FPR*	*F-measure*
CRFs	HIV1_01_AE	1000	0.989	0.000	0.993	0.007	0.000	0.014	0.956	0.001	0.975
	HIV1_02_AG	1000	0.952	0.002	0.967	0.000	0.000	0.000	0.853	0.005	0.897
	HIV1_06_cpx	698	0.924	0.000	0.958	0.938	0.000	0.965	0.927	0.003	0.943
	HIV1_07_BC	1000	0.977	0.000	0.988	0.988	0.000	0.993	0.982	0.002	0.980
	HIV1_08_BC	399	0.965	0.000	0.981	0.990	0.000	0.994	0.972	0.001	0.970
	HIV1_11_cpx	58	0.828	0.000	0.906	0.690	0.000	0.816	0.897	0.006	0.588
	HIV1_12_BF	222	0.860	0.000	0.925	0.374	0.000	0.544	0.932	0.008	0.807
	Mean		0.928	0.000	0.960	0.570	0.000	0.618	0.931	0.004	0.880
Pure subtypes	HIV1_A	1000	0.966	0.001	0.980	0.968	0.106	0.654	0.891	0.006	0.917
	HIV1_B	1000	0.995	0.001	0.993	0.945	0.000	0.970	0.817	0.007	0.866
	HIV1_C	1000	0.990	0.001	0.991	0.997	0.000	0.997	0.912	0.003	0.942
	HIV1_D	1000	0.938	0.000	0.968	0.911	0.000	0.953	0.892	0.010	0.899
	HIV1_F	1000	0.927	0.000	0.962	0.970	0.000	0.985	0.914	0.003	0.940
	HIV1_G	1000	0.915	0.001	0.952	0.929	0.007	0.931	0.778	0.003	0.860
	Mean		0.955	0.001	0.974	0.953	0.019	0.915	0.867	0.005	0.904

This table contains TPR, FPR and *F-measure* of COMET, REGA and CASTOR on the prediction of HIV-1 M pure subtypes and CFRs *pol* fragments. The shown classes belong to the common subtypes sampling. The CASTOR model used in this evaluation is PMSHIV03

Even though CASTOR is not a specific HIV-1 classifier, it competes with the most powerful methods in HIV-1. Unlike COMET and REGA, CASTOR provides an easy way of performing several types of classification (see Table 2). It also has no restriction on the size of data and is time efficient. Hence, we completed the analysis by performing a test on the whole Los Alamos HIV dataset (without the training sequences of the three methods). For CGs (3 778 instances), CASTOR completes the test in 1 min 59 s with an accuracy of 91.2%. While for the *pol* fragments (119 005 instances), it requires 20min10s with an accuracy of 85.41%. It shows that CASTOR takes 0.01s to process a sequence that is far more efficient than the time results indicated in [2] for REGA (28s/sequence), but 10-fold less efficient than COMET (0.001s/sequence) [2]. Furthermore, due to size issues, it is not possible to perform such large analyses in actual version of COMET server. Overall, CASTOR highlights a good accuracy on the classification of the three studied viruses. However this accuracy is slightly lower than specific virus predictors as shown previously. But it exhibits more analysis capacity, permitting several and highly accurate set of classifications. As shown in Table 2, this accuracy is higher than 90% for almost all studies except for comparing HIV-1 M pure subtypes vs CRFs. For less complex genomes such as HPV and HBV, the mean of weighted *F-measures* is higher than 0.912. CASTOR will allow to increase the class representatives, to add or remove classes and also to benchmark several types of classification. For viruses without existing predictors, it could accurately cover the needs as it is for HPV, instead of relying on the similarity sequence search such as BLAST [3] or USEARCH [4]. Sequence search is generally not recommended for subtyping since it will not allow the identification of novel forms, it cannot also aggregate common attributes of a class while predicting [2, 4].

### CASTOR web platform

CASTOR is available as a public web platform. It is composed of four main applications. (1) **CASTOR-build** allows users to create and train new classifiers from a set of labeled virus sequences. It contains default parameters and advanced options letting users to customize the classifier parameters. It can be used also to update the parameters or input sequences of an already built classifier. The constructed classifiers can be saved in an exportable file locally or published to the community via CASTOR-database described below. (2) **CASTOR-optimize** constructs improved classifiers. Unlike CASTOR-build that allows users to define metrics, algorithms and feature selection techniques, it assesses all combinations of the classification parameters and provides the best fitting classifier according to the input data. (3) **CASTOR-predict** is the kernel application that allows users to annotate viral sequences according to a chosen classifier. Also, it serves as an evaluation module for classifiers with labeled test sets. The results are provided with enriched graphics and performance measures (4) **CASTOR-database** is a public database of classifiers which allows the community to share their expertise and models. It facilitates experiment reproducibility and model refinement. A characteristic viewer and a search engine of the published classifiers are also implemented. Hence, from the interface of CASTOR-database, users can download, reuse, update and comment the classifiers. To the best of our knowledge, CASTOR constitutes the first RFLP-based prediction platform for the classification of viral sequences.

## Conclusion

In this paper, we have shown that RFLP has a great performance in large scale sequence classification. We also provide CASTOR, the first viral sequence classification platform based on RFLP. We claim that CASTOR can perform well for different types of viruses (Group I, Group VI and Group VII) with mean of weighted *F-measures* > 0.900 in most cases (see Table 1). In the future, we will attempt to increase the performance by modelling the boundaries of the classes and including an *open-set* approach to deal with instances from unknown classes. The CASTOR platform implements several metrics and classifiers, allowing generic and diverse analyses within the same environment. CASTOR allows the storage of models enabling reproducible experiments and open data access. Even though CASTOR is scaled for viruses, it can be used and extended easily for other types of organisms, including whole genome and partial sequences. In the future, more models will be included, in particular those specialized in less studied organisms and/or without dedicated tools. In addition, scientists could add their tuned models helping CASTOR to enhance the predictions. We will also optimize the platform to allow other types of classification such as functional, disease related and geographical classifications. Hence, CASTOR could quickly become a reference in comparative genomics focusing on various types of sequence classification.

## Additional file

Additional file 1 Supplementary data. This PDF file contains supplementary Table S1 and supplementary Figures S1–S4. (PDF 1638 kb)

## Abbreviations

ADA: Adaboost
BAG: Bagging
DNA: Deoxyribonucleic acid
FN: False negative
FP: False positive
FPR: False positive rate
HBV: Hepatitis B viruses
HIV: Human immunodeficiency viruses
HPV: Human papillomaviruses
IBK: K-nearest neighbors
J48: C4
5 decision tree; MDS: Multidimensional scaling NBA: Naive Bayes classifier
RFLP: Restriction fragment length polymorphism
RFT: Random forests
RMS: Root mean square
SVM: Support vector machine
TN: True negative
TP: True positive
TPR: True positive rate
