# Supplementary material for: A machine learning approach for viral genome classification
Source: BMC Bioinformatics. 2017 Apr 11;18:208. doi: 10.1186/s12859-017-1602-3 (PMC5387389; doi:10.1186/s12859-017-1602-3)
Supplement: Additional file 1 — Supplementary data. This PDF file contains supplementary Table S1 and supplementary Figures S1–S4. (PDF 1638 kb) [file 12859_2017_1602_MOESM1_ESM.pdf]

ADDITIONAL FILE 1

Supplementary data: A machine learning approach for viral genome classification

Mohamed Amine Remita, Ahmed Halioui, Abou Abdallah Malick Diouara, Bruno Daigle, Golrokh Kiani and Abdoulaye Baniré Diallo*

*Correspondence: diallo.abdoulaye@uqam.ca
Laboratoire de bioinformatique, département d'informatique, Université du Québec à Montréal, P.O. Box 8888 Downtown Station, H3C 3P8, Montreal, Qc, Canada

Table S1 Learning algorithms.

Algorithm type	Algorithm	Weka module	Options	Acronym
Symbolic	C4.5 decision tree	weka.classifiers.trees.J48	-C 0.25 -M 2	J48
	Random forests	weka.classifiers.trees.RandomForest	-I 10 -K 0 -S 1 -num-slots 1	RFT
Statistical	Naive bayes	weka.classifiers.bayes.NaiveBayes	NaiveBayes	NBA
	Support vector machine	weka.classifiers.functions.LibSVM	-S 0 -K 0 -D 3 -G 0.0 -R 0.0 -N 0.5 -M 40.0 -C 1.0 -E 0.001 -P 0.1 -model /home/hpvs/weka-3-7-10 -seed 1	SVM
	K-nearest neighbours	weka.classifiers.lazy.IBk	-K $K -W 0 -A "weka.core.neighboursearch.LinearNNSearch -A "weka.core.EuclideanDistance -R first-last""	IBK
Ensemble	AdaBoost	weka.classifiers.meta.AdaBoostM1	-P 100 -S 1 -I 10 -W weka.classifiers.trees.J48 - -C 0.25 -M 2	ADA
	Bagging	weka.classifiers.meta.Bagging	-P 100 -S 1 -num-slots 1 -I 10 -W weka.classifiers.trees.J48 - -C 0.25 -M 2	BAG

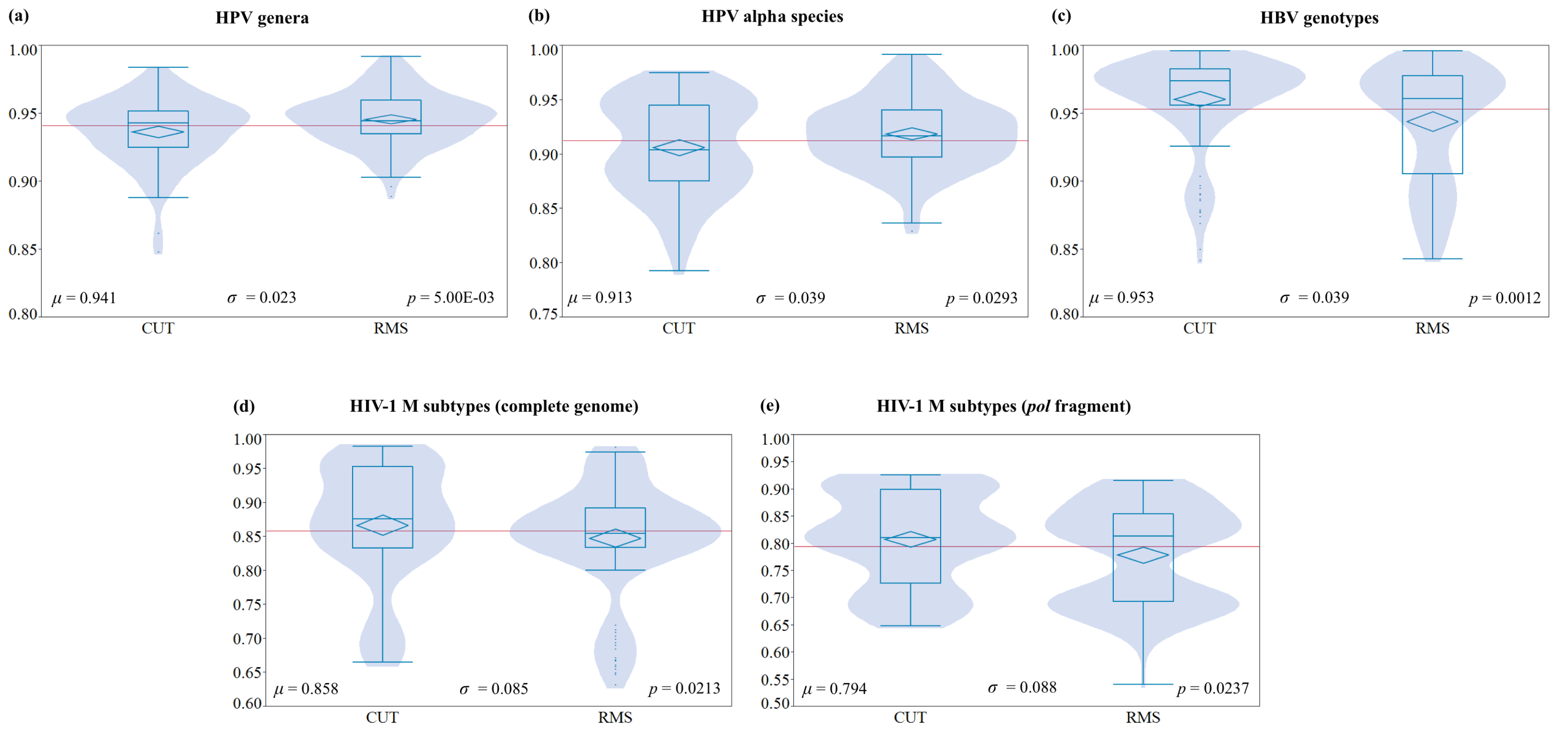

Figure S1 Comparison of the weighted *F-measure* distribution according to *CUT* and *RMS* computed from the simulation study of the 280 experiments for **(a)** HPV genera, **(b)** HPV alpha species, **(c)** HBV genotypes, **(d)** HIV-1 M subtype complete genomes and **(e)** HIV-1 M subtype *pol* fragments. μ, σ are the mean and the standard deviation of the overall weighted *F-measures*. *p* is the *p-value* determining the statistically significance of the weighted *F-measure* mean differences among all the experiments. This *p-value* is computed with the Wilcoxon/Kruskal-Wallis test.

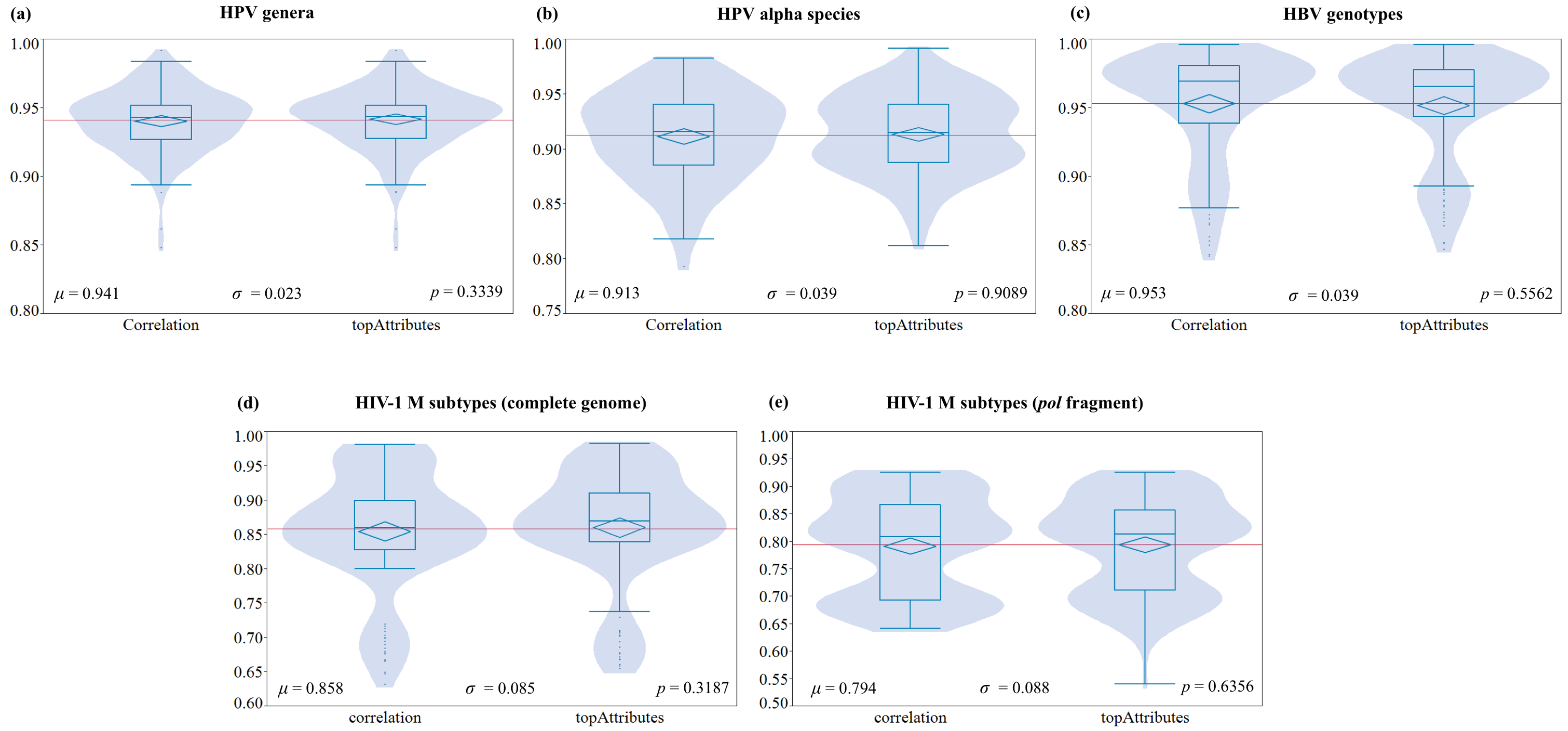

Figure S2 Comparison of the weighted *F-measure* distribution according to *topAttributs* and *correlation* computed from the simulation study of the 280 experiments for **(a)** HPV genera, **(b)** HPV alpha species, **(c)** HBV genotypes, **(d)** HIV-1 M subtype complete genomes and **(e)** HIV-1 M subtype *pol* fragments. μ, σ are the mean and the standard deviation of the overall weighted *F-measures*. *p* is the *p-value* determining the statistically significance of the weighted *F-measure* mean differences among all the experiments. This *p-value* is computed with the Wilcoxon/Kruskal-Wallis test.

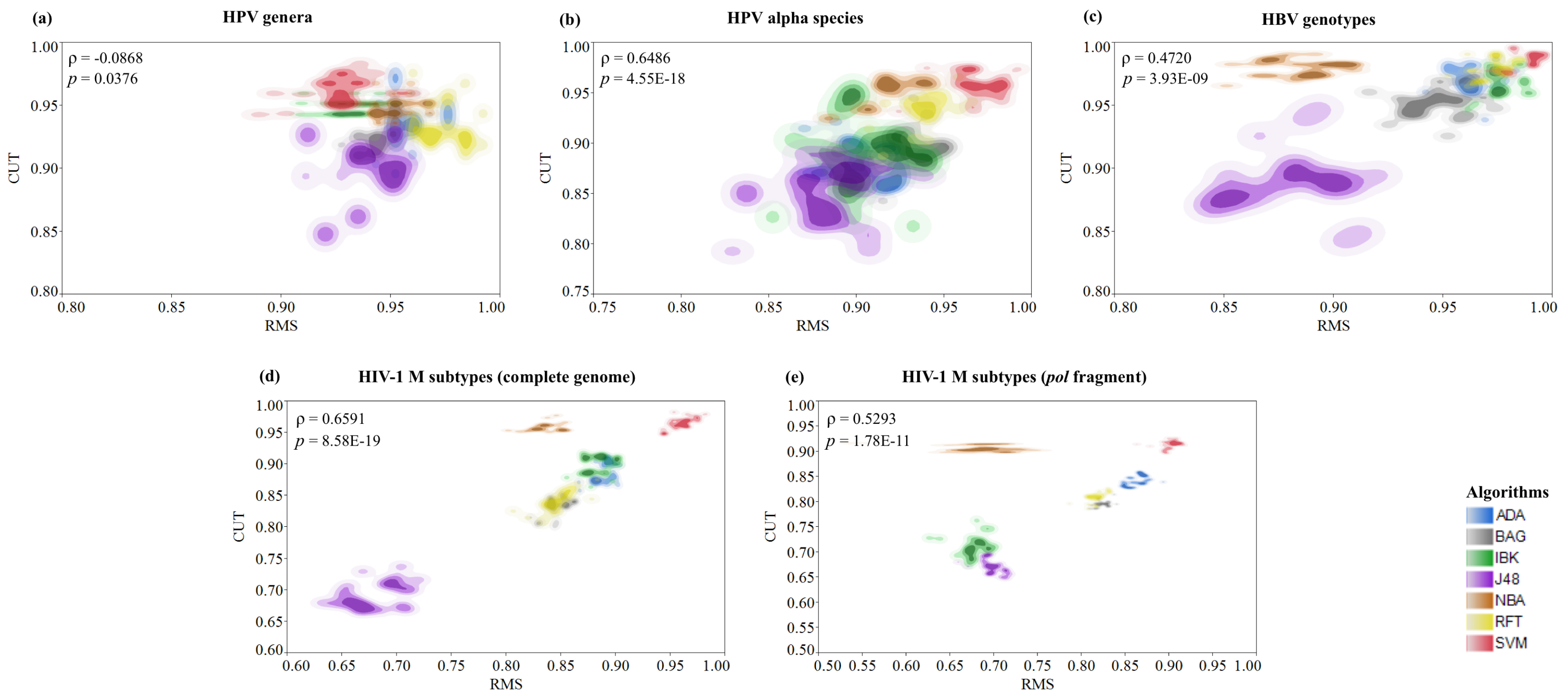

Figure S3 CUT/RMS weighted *F-measure* correlation computed from the simulation study of the 280 experiments for **(a)** HPV genera, **(b)** HPV alpha species, **(c)** HBV genotypes, **(d)** HIV-1 M subtype complete genomes and **(e)** HIV-1 M subtype *pol* fragments. The weighted *F-measure* values are clustered according to the seven machine learning algorithms indicated in the legend. The values in top left corner of each figures correspond to the Spearman correlation score and the associated *p-value*.

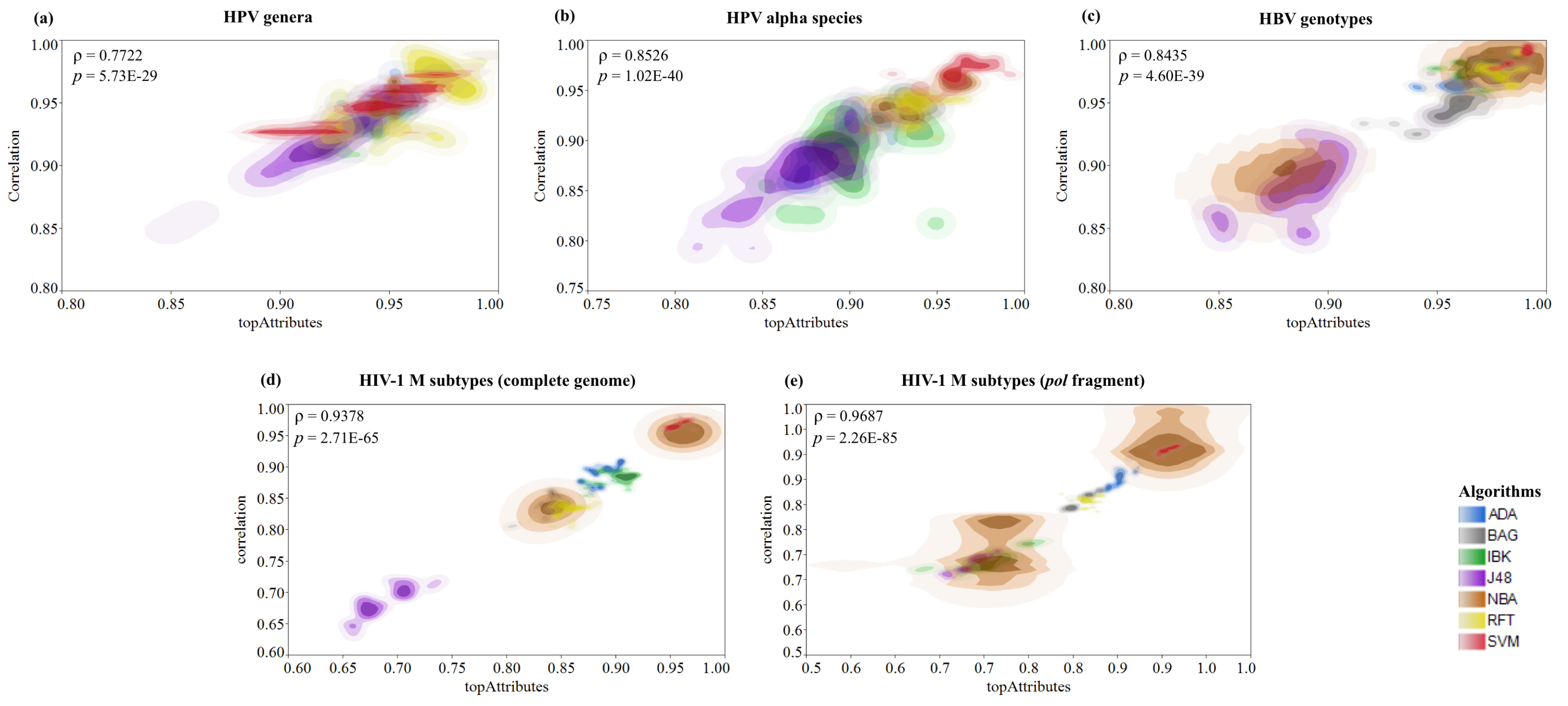

Figure S4 *Correlation/topAttributes* Spearman correlations based on the weighted *F-measures* computed from the simulation study of the 280 experiments for **(a)** HPV genera, **(b)** HPV alpha species, **(c)** HBV genotypes, **(d)** HIV-1 M subtype complete genomes and **(e)** HIV-1 M subtype *pol* fragments. The weighted *F-measure* values are clustered according to the seven machine learning algorithms indicated in the legend. The values in top left corner of each figures correspond to the Spearman correlation score and the associated *p-value*.
